# The Oral–Brain Axis: A Unified Framework Linking Trigeminal Sensorimotor Dysfunction, Chronic Stress, Neuroinflammation, and Neurodegeneration

**DOI:** 10.3390/ijms27177597

**Published:** 2026-08-25

**Authors:** Hiroki Toyoda

**Affiliations:** Department of Physiology, School of Dentistry, Aichi Gakuin University, Nagoya 464-8650, Japan; toyoda@dpc.agu.ac.jp; Tel.: +81-52-757-6741

**Keywords:** oral–brain axis, mesencephalic trigeminal nucleus, predictive coding, locus coeruleus, asparagine endopeptidase, neurodegeneration

## Abstract

Neurodegenerative diseases such as Alzheimer’s disease (AD) and Parkinson’s disease (PD) develop over decades, yet their earliest pathogenic drivers remain poorly understood. Epidemiological and experimental animal studies suggest that disturbances in oral sensorimotor regulation, particularly within trigeminal proprioceptive pathways, may contribute to neural dysfunction long before clinical symptoms emerge. The mesencephalic trigeminal nucleus (MesV), the only primary sensory neuron population located entirely within the central nervous system (CNS), links oral proprioception with brainstem and forebrain networks. Chronic occlusal mismatch, impaired mastication, sleep bruxism, and sleep-disordered breathing may generate persistent sensorimotor prediction errors that destabilize MesV-centered circuits and subsequently recruit the locus coeruleus (LC), the brain’s principal noradrenergic stress nucleus. This review proposes an oral–brain axis model in which chronic MesV-related prediction error signaling engages LC-dependent stress systems, leading to neuroimmune activation, locus coeruleus–asparagine endopeptidase (LC-AEP) pathway engagement, and downstream proteinopathic processes. Sustained LC activity may facilitate microglial priming, reactive astrocytosis, and neuroinflammatory signaling, creating conditions that favor LC-AEP pathway activation and downstream tau pathology. Epidemiological studies associate tooth loss, reduced occlusal support, and impaired mastication with increased dementia risk, while experimental models of prodromal PD demonstrate early trigeminal sensory-processing abnormalities preceding motor symptoms. Together, these findings support the hypothesis that chronic disturbances in oral sensorimotor homeostasis may increase neurodegenerative vulnerability. This framework identifies potential biomarkers and preventive targets, suggesting that modulation of oral function and neuroimmune pathways may help reduce neurodegenerative risk before irreversible neuronal loss occurs.

## 1. Introduction

Neurodegenerative diseases such as Alzheimer’s disease (AD) and Parkinson’s disease (PD) represent major global health challenges, largely because their pathological processes begin decades before clinical symptoms become apparent. By the time cognitive or motor deficits become clinically detectable, extensive synaptic dysfunction, neuronal loss, and pathological protein aggregation have often already occurred, substantially limiting the efficacy of disease-modifying interventions. Consequently, increasing emphasis has been placed on identifying upstream mechanisms and prodromal biomarkers that contribute to disease initiation rather than merely reflecting downstream pathological consequences [1,2,3,4].

Although oral dysfunction has traditionally been regarded as a secondary manifestation of aging or neurological disease, accumulating epidemiological and experimental evidence suggests a more complex bidirectional relationship between oral health and brain function. Tooth loss, impaired mastication, occlusal abnormalities, and oral sensory disturbances have been consistently associated with cognitive decline, dementia, and AD risk [5,6,7,8]. These associations have been reported across diverse populations and study designs, raising the possibility that oral dysfunction may contribute to biological processes influencing brain aging and neurodegenerative vulnerability [7,8,9]. Although periodontal inflammation is a recognized contributor to neurodegenerative risk through systemic inflammatory mechanisms, the present review focuses primarily on oral sensorimotor pathways.

The concept of the oral–brain axis provides a useful framework for understanding this relationship. Oral sensory input is not merely involved in feeding behavior but constitutes a continuous stream of proprioceptive, tactile, nociceptive, and chemosensory information transmitted through trigeminal pathways to distributed neural networks, including the insular cortex, amygdala, hippocampus, hypothalamus, and locus coeruleus (LC) [7,10,11,12]. Through these extensive connections, trigeminal signaling contributes to sensorimotor integration, autonomic regulation, emotional processing, arousal, learning, and adaptive behavior [7,10,11,12,13,14]. 

Among trigeminal structures, the mesencephalic trigeminal nucleus (MesV) occupies a uniquely important position. MesV neurons are the only primary sensory neurons whose cell bodies reside entirely within the central nervous system (CNS), placing them at the interface between peripheral oral proprioception and central neural regulation [11,15]. These neurons continuously encode jaw position, occlusal force, and masticatory dynamics and contribute directly to the regulation of masticatory motor output [11,15]. Consequently, disturbances in oral sensory input, including occlusal instability and alterations in the vertical dimension of occlusion (VDO), may impose persistent computational demands on sensorimotor circuits and challenge the maintenance of neural homeostasis [7,10,16,17,18,19]. According to predictive-coding models [20], the brain continuously generates internal predictions regarding the sensory consequences of movement and adjusts behavior based on discrepancies between expected and actual sensory feedback [18,19]. Chronic occlusal mismatch may therefore function as a persistent source of prediction error, contributing to increased demands on MesV-centered proprioceptive networks and vulnerability to maladaptive neural plasticity [17,21].

A second emerging theme is the convergence between oral dysfunction and chronic stress. Conditions such as altered occlusion, masticatory insufficiency, sleep bruxism, and sleep-disordered breathing repeatedly engage neural systems involved in stress regulation [17,22,23]. Of particular relevance is the LC, the principal noradrenergic nucleus of the brain, which plays essential roles in arousal, attention, adaptive behavior, and stress responsiveness [13,14] and undergoes early pathological alterations in both AD and PD [24,25,26]. Chronic engagement of LC-dependent stress pathways may therefore represent an important mechanism through which oral dysfunction exerts widespread effects on brain function [13,14,23]. 

Experimental animal models and biochemical studies have further implicated asparagine endopeptidase (AEP; also known as delta-secretase) as a potential molecular link between chronic stress and neurodegenerative pathology. AEP cleaves tau into aggregation-prone fragments and contributes to the propagation of tau pathology in AD models [27,28]. Moreover, specific evidence from experimental animal models suggests that chronic stress and LC dysfunction may promote AEP activation, thereby linking stress-responsive neural circuits with mechanisms of proteinopathy and neurodegeneration [29,30].

A further emerging concept relevant to the oral–brain axis is the growing recognition that neurodegeneration is strongly influenced by neuroimmune and glial mechanisms [31,32,33,34]. Microglia and astrocytes play essential roles in maintaining CNS homeostasis, regulating synaptic function, neurotransmitter balance, and metabolic support [31,32,33,34]. Under chronic stress or pathological conditions, however, these glial populations undergo functional reprogramming characterized by increased inflammatory signaling and impaired homeostatic regulation [31,32].

Importantly, chronic stress and neuroinflammation are increasingly recognized as interconnected processes [35,36]. Sustained activation of stress-responsive circuits, particularly those involving the LC, can influence glial function and promote microglial priming and reactive astrocytosis [34,36]. Consequently, chronic oral sensorimotor dysfunction may contribute to neurodegenerative vulnerability not only through altered neural activity but also through downstream neuroimmune mechanisms [24,31,32,37]. This concept provides a bridge between oral sensorimotor disturbances and the neuroinflammatory pathways discussed later in this review.

This review proposes that dysfunction within trigeminal sensory networks, particularly those involving MesV and LC-associated pathways, represents an underappreciated contributor to neurodegenerative disease. By integrating evidence from occlusal mismatch, masticatory dysfunction, predictive-coding theory, prodromal Parkinsonian sensory-gating abnormalities, chronic stress biology, sleep bruxism, obstructive sleep apnea (OSA), neuroinflammation, and AEP-dependent tau pathology, this review outlines a unifying oral–brain axis hypothesis in which disturbances of oral sensorimotor homeostasis may contribute to maladaptive neural plasticity, stress-system dysregulation, neuroimmune activation, and ultimately neurodegeneration. Among the various components of the trigeminal system, MesV is uniquely positioned to integrate oral proprioceptive signals with central neuromodulatory networks, making it a plausible upstream node of the proposed oral–brain axis.

## 2. Hierarchical Neural Control of Mastication

### 2.1. From Occlusal Mismatch to MesV Dysfunction

Experimental models such as soft-diet feeding, molar extraction, and reduced occlusal support have long demonstrated that diminished masticatory activity impairs hippocampal neurogenesis, spatial learning, memory performance, and synaptic plasticity [7,10,16,38]. However, these traditional models introduce potential confounding factors, including inflammation, altered craniofacial biomechanics, and nutritional changes, making it difficult to isolate the specific contribution of altered sensory input [17]. To overcome these limitations, controlled VDO-manipulation paradigms and sensorimotor perturbation models have selectively perturbed oral sensorimotor regulation while preserving dentition and nutritional status [17,29]. Manipulation of the VDO is particularly informative because it maintains masticatory activity while introducing a persistent mismatch between predicted and actual sensory feedback [18,19,39]. This mismatch disrupts the equilibrium between predicted and actual proprioceptive input, requiring continuous recalibration of sensorimotor circuits [17,18,19].

MesV neurons play a central role in this process because they constitute the principal proprioceptive component of the trigeminal system [11,21]. Through monosynaptic and oligosynaptic projections to trigeminal motoneurons, MesV neurons enable rapid adjustments in jaw-muscle activation and force generation [11,15,21]. Toyoda et al. demonstrated this concept using a guinea pig model, where the VDO was chronically altered by applying dental acrylic caps to the bilateral mandibular molars [39]. This experimental manipulation selectively introduced a persistent sensorimotor prediction error during mastication while fully preserving active dentition and normal nutritional intake [39]. Behavioral assessments revealed that this chronic VDO increase induced progressive, duration-dependent impairments in spatial learning and memory performance [39]. These findings strongly suggest that sustained oral sensorimotor mismatch alone, independent of tooth loss or malnutrition, can function as a chronic form of biological stress that disrupts cognitive networks [13,14,23,39]. However, a key limitation of this behavioral model is that it does not provide direct electrophysiological or morphological evidence of cellular dysfunction within the MesV-LC pathway itself. Further integrative studies are required to elucidate the precise brainstem synaptic mechanisms linking peripheral occlusal mismatch to downstream neuromodulatory dysregulation.

### 2.2. MesV Vulnerability and Channelopathy

A critical unresolved question concerns the neural pathway through which localized oral sensorimotor disturbances influence broader cognitive and neuromodulatory systems. The MesV has emerged as a compelling candidate because of its unique anatomical, developmental, and electrophysiological characteristics [11,21]. This distinctive anatomical arrangement places it at a strategic interface between peripheral proprioceptive signaling and central neural regulation [11]. In addition to their unique anatomical location within the CNS, MesV neurons display developmental and electrophysiological characteristics that may increase vulnerability to chronic stress and aging-related pathology. In addition, MesV is located in close proximity to several brainstem nuclei involved in arousal and stress regulation, including the LC, raising the possibility that pathological processes affecting the brainstem may also influence MesV function [11,13,14].

MesV vulnerability may also reflect developmental factors. These neurons undergo prolonged postnatal maturation, particularly during the transition from suckling to mastication, a period during which activity-dependent plasticity plays a major role in shaping sensorimotor circuits [11,40,41]. Functional refinement during this developmental window contributes to the establishment of long-term patterns of excitability and proprioceptive processing [11,21]. Consequently, early-life sensory deprivation, abnormal masticatory experience, or chronic stress may produce subtle alterations in circuit organization that remain latent for decades before interacting with aging-related and neurodegenerative processes [11,21]. In addition to their distinctive anatomical and developmental features, MesV neurons possess highly specialized electrophysiological properties that are essential for the stable encoding of proprioceptive information. Accurate representation of jaw position, occlusal force, and masticatory dynamics depends on precise regulation of membrane excitability [11,21]. This regulation is achieved through the coordinated activity of multiple ion-channel systems, including TASK1 (TWIK-related acid-sensitive K^+^ channel 1) and TASK3 leak potassium channels [42,43], hyperpolarization-activated cyclic nucleotide-gated channels [21,44], and voltage-gated sodium channels [21,44]. Together, these channels maintain firing stability and enable rapid adaptation to changing sensorimotor demands.

Importantly, several of these mechanisms are particularly susceptible to disturbances in intracellular calcium homeostasis, which is increasingly recognized as an early pathogenic event in neurodegenerative disorders [45,46]. Calcium dysregulation can alter channel function, destabilize membrane excitability, and disrupt the precise temporal patterns of neuronal activity required for effective proprioceptive signaling. As a consequence, ion-channel dysfunction may impair sensory encoding within MesV circuits [11,21]. Such impairment has the potential to initiate a maladaptive cycle in which abnormal motor output generates altered sensory feedback, creating further demands on sensorimotor recalibration and increasing network instability [18,19].

Viewed collectively, these observations suggest that MesV neurons possess a unique combination of anatomical accessibility, developmental sensitivity, and electrophysiological specialization that may render them particularly vulnerable to chronic oral sensorimotor disturbances. Although direct evidence linking MesV dysfunction to neurodegenerative disease remains limited, the available findings support the hypothesis that channelopathy within MesV neurons could represent an early mechanistic link between oral dysfunction and broader neural instability. This possibility provides a conceptual basis for the MesV-centered model proposed in the following section.

### 2.3. Unified MesV Model

**Figure 1:**
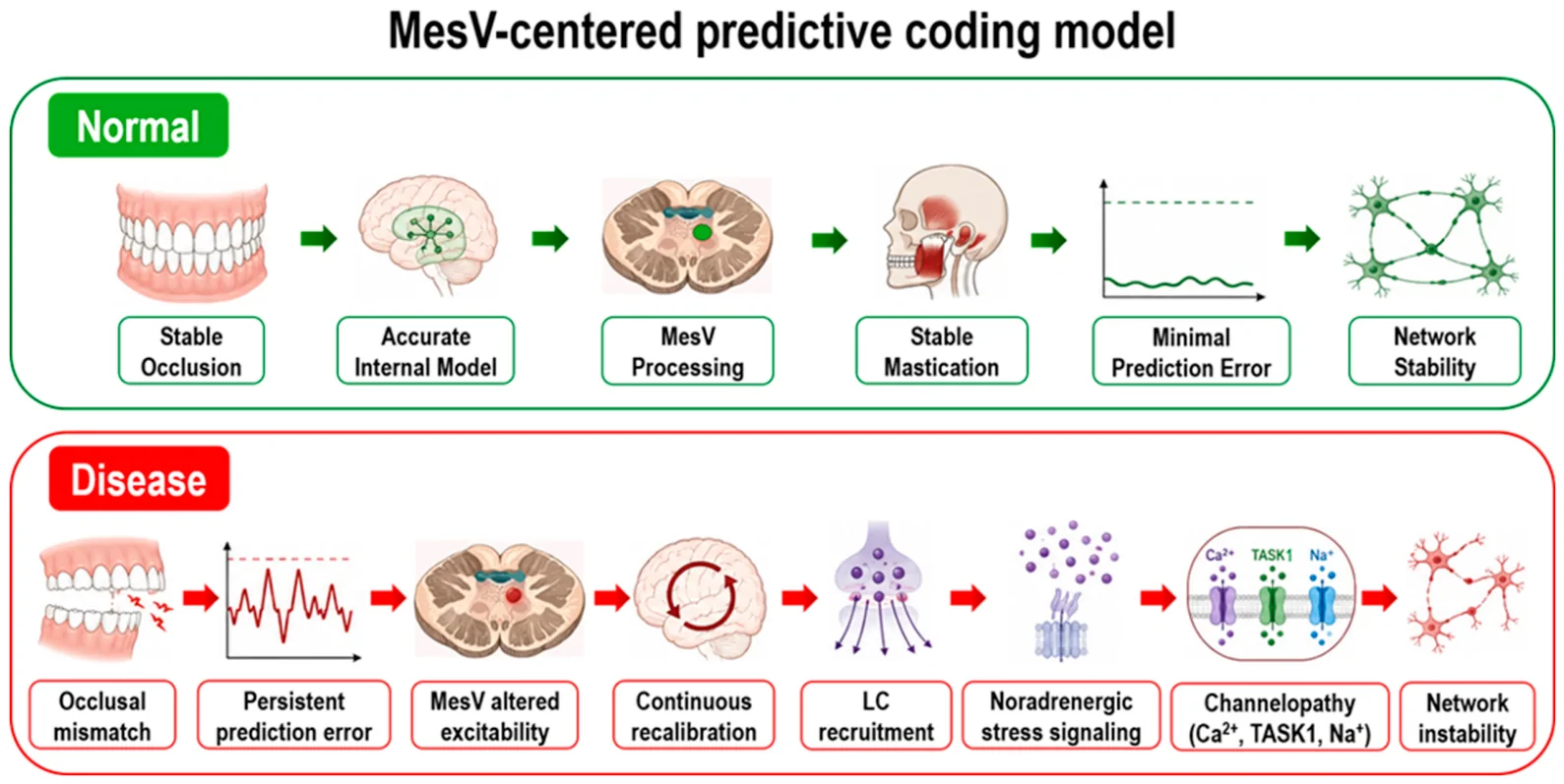
MesV-centered predictive-coding model of occlusal homeostasis and disease. Proposed predictive-coding framework centered on the trigeminal mesencephalic nucleus (MesV). Under physiological conditions, stable occlusion enables accurate sensorimotor internal models, resulting in efficient mastication with minimal prediction error and maintenance of network stability. In contrast, chronic occlusal mismatch may generate persistent prediction errors that require continuous sensorimotor recalibration within MesV-centered circuits. Prolonged processing demands may contribute to altered neuronal excitability, calcium dysregulation, and ion-channel dysfunction involving TASK1 (TWIK-related acid-sensitive K^+^ channel 1) and voltage-gated sodium channels. Through downstream brainstem pathways, these changes may engage locus coeruleus (LC)-related stress systems and promote progressive network instability.

Chronic occlusal mismatch generates persistent discrepancies between predicted and actual sensory feedback [17,18,19]. MesV-centered proprioceptive networks detect these discrepancies and may contribute to compensatory recalibration [11,21]. Although initially adaptive, prolonged compensation increases metabolic demand [17,18] and destabilizes membrane excitability through calcium dysregulation and ion-channel dysfunction [45,47,48,49]. These alterations propagate via polysynaptic pathways to downstream neuromodulatory systems, particularly the LC [13,14,24], which regulates arousal, attention, stress responsiveness, and memory formation [13,14]. The resulting predictive-coding failure promotes maladaptive neural plasticity and progressive network instability [18,19]. Collectively, these findings support a MesV-centered predictive-coding model in which chronic occlusal mismatch generates persistent sensorimotor prediction errors, leading to continuous recalibration, neuromodulatory dysregulation, and progressive network instability. A schematic overview of the proposed mechanism is shown in Figure 1.

### 2.4. MesV-LC Coupling: A Bridge Between Oral Sensorimotor Networks and Stress Regulation

A critical question is how localized disturbances in oral sensorimotor processing influence widespread neural networks involved in cognition and neurodegeneration. One potential answer lies in the functional coupling between trigeminal pathways and the LC. The LC receives convergent inputs from multiple sensory systems, including trigeminal pathways that continuously monitor the oral and facial environment [11,13,14]. Although direct monosynaptic connections between MesV neurons and the LC have not been established, MesV-related signals may influence LC activity indirectly through trigeminal nuclei, reticular formation circuits, and parabrachial pathways that are themselves connected with the LC [11,13,14]. Conversely, recent electrophysiological evidence demonstrates that LC activity can directly modulate MesV neuronal function through noradrenergic volume transmission. Dual whole-cell recordings from LC and MesV neurons revealed that LC activation suppresses hyperpolarization-activated currents in MesV neurons via α2-adrenergic receptor-dependent mechanisms, providing a physiological basis for bidirectional LC-MesV interactions [50].

The LC functions as a central detector of unexpected sensory events and allocates neural resources to conditions requiring adaptive responses [13,14]. Under conditions of stable occlusion, sensorimotor prediction errors are presumed to remain minimal and LC activation is relatively limited [18,19]. Chronic occlusal mismatch, however, generates persistent prediction errors that repeatedly recruit LC activity. Over time, transient adaptive responses may shift toward sustained stress-related activation [14,18,19]. Excessive LC activity propagates downstream through noradrenergic volume transmission [13,14]. Unlike classical synaptic transmission, norepinephrine diffuses through the extracellular space, modulating neuronal excitability across broad regions of the brainstem [13,14]. Under chronic stress, dysregulated noradrenergic signaling alters membrane excitability [13,14,29], impairs proprioceptive encoding [11,21], amplifies sensory prediction errors, and reinforces maladaptive motor output. Over time, these changes may progressively destabilize MesV-centered sensorimotor networks, potentially creating a self-reinforcing feedforward loop [13,14,29] in which trigeminal dysfunction contributes to increased LC activity, while altered LC output may further aggravate trigeminal network instability [11,13,14,24,25,26,29].

Because the LC undergoes early pathological changes in both AD and PD [24,25,26], chronic interactions between trigeminal sensorimotor dysfunction and LC-related neuromodulatory systems may represent a biologically plausible mechanism through which long-standing oral sensory dysfunction contributes to broader neuromodulatory decline and neurodegenerative vulnerability. Although LC dysregulation has been implicated in molecular pathways associated with tau pathology, including AEP-related mechanisms, the detailed LC–AEP cascade is described in Section 6.

## 3. Human Evidence Supporting the Oral–Brain Axis

### 3.1. Periodontal Inflammation and Neurodegeneration

Although the present review primarily focuses on oral sensorimotor pathways, periodontal inflammation represents another important mechanism linking oral dysfunction and brain health. Periodontitis is a chronic inflammatory disease and a major cause of tooth loss in older adults [51,52]. Increasing evidence suggests that periodontal disease may contribute to neurodegeneration through systemic inflammatory pathways distinct from those proposed for trigeminal sensorimotor dysfunction [31,32,51,52].

Periodontal pathogens and their virulence factors, including lipopolysaccharides derived from Porphyromonas gingivalis, can enter the systemic circulation and induce chronic low-grade inflammation [51,52,53]. Elevated inflammatory mediators such as interleukin-1β, interleukin-6, and tumor necrosis factor-α may disrupt blood–brain barrier integrity, activate microglia, and promote neuroinflammatory responses associated with neurodegenerative vulnerability [31,32,51,52,54]. Experimental and clinical studies further suggest links between periodontal inflammation and amyloid-β accumulation, tau pathology, and neuroimmune dysregulation [51,52].

Thus, oral health may influence brain function through at least two partially overlapping mechanisms: (1) inflammatory pathways associated with periodontal disease and tooth loss [51,52], and (2) sensorimotor pathways involving trigeminal networks, MesV dysfunction, and LC-dependent stress responses [7,11,13,14,21]. While these mechanisms may interact, the present review focuses on the second pathway because of its potential role in predictive-coding dysfunction, chronic stress signaling, and neurodegenerative vulnerability [7,11,13,14,18,19,21,37].

### 3.2. Tooth Loss and Dementia Risk

A substantial body of epidemiological research demonstrates that oral dysfunction, particularly tooth loss, is strongly associated with cognitive decline and dementia risk [5,7,8,9]. Large population-based cohort studies consistently show that individuals with fewer remaining teeth have a significantly higher likelihood of developing dementia and AD [5,8,9]. Meta-analyses further indicate a dose-dependent relationship, whereby increasing tooth loss is associated with progressively greater cognitive impairment and dementia risk [5,28]. Importantly, these associations persist after adjustment for socioeconomic status, education, nutritional factors, and medical comorbidities, suggesting that tooth loss is not merely a marker of aging or social disadvantage [8,9]. Rather, it may reflect disturbances in masticatory input [7,10,17], trigeminal signaling [11,21], systemic inflammation [51,52], and neuromodulatory regulation [13,14] that collectively contribute to accelerated brain aging and increased neurodegenerative vulnerability [5,7,8,9,28]. Within the oral–brain axis framework, tooth loss can be viewed as a reduction in proprioceptive and mechanosensory input to trigeminal pathways [7,11]. Because these pathways influence hippocampal function [7,10], stress regulation [14,23], and neuromodulatory tone [13,14], diminished oral sensory input may increase susceptibility to cognitive decline and neurodegenerative disease [7,13].

Beyond cognitive deterioration, emerging human and experimental evidence indicates that oral dysfunction is also closely linked to non-cognitive and neuropsychiatric symptoms in patients with AD [51,52]. Impaired mastication, tooth loss, and secondary nutritional decline are increasingly associated with the severity of behavioral and psychological symptoms of dementia, such as agitation, apathy, anxiety, and depression [51,52]. Mechanistic studies further suggest that molar loss and reduced trigeminal sensory feedback can diminish monoaminergic neurotransmission, particularly hippocampal serotonin levels, which may contribute to aggressive behavior and emotional instability in AD pathology. Given that the oral–brain axis model involves critical integration with the LC and stress-responsive systems, chronic oral sensorimotor deficits may also disrupt sleep–wake regulation and autonomic balance, thereby contributing significantly to the complex matrix of non-cognitive symptoms that characterize clinical AD progression [7,13,14].

### 3.3. Clinical and Epidemiological Evidence in Humans

Mastication is increasingly recognized as a profound regulator of brain function rather than merely a mechanical component of food processing [7,10]. Functional neuroimaging studies demonstrate that chewing activates widespread cortical and subcortical networks, including the sensorimotor cortex, insular cortex, hippocampus, cerebellum, and prefrontal cortex [10,55,56]. These activations reflect the integration of proprioceptive, tactile, and mechanosensory information transmitted through trigeminal pathways and associated sensorimotor networks [11,15].

Clinical investigations further indicate that impaired masticatory performance is significantly associated with deficits in specific cognitive domains, such as poorer memory, executive function, and information-processing speed in older adults [6,7,57]. Conversely, the preservation of occlusal support, bite force, and masticatory efficiency strongly correlates with better cognitive performance and quality of life [6,7,16,57].

Longitudinal investigations provide even stronger evidence for a temporal relationship between oral dysfunction and cognitive decline [5,8,9,57]. Prospective cohort studies demonstrate that baseline oral dysfunction, including tooth loss, reduced occlusal support, diminished bite force, and impaired chewing ability, reliably predicts future cognitive decline and increased dementia risk [5,6,8,9,57]. Importantly, these longitudinal associations often precede clinical diagnosis by several years, suggesting that oral dysfunction may serve as an early biomarker of neurodegenerative vulnerability rather than merely reflecting established disease [5,8,9,57]. While direct causality remains unproven in humans, the temporal sequence is consistent with the hypothesis that oral dysfunction contributes to disease progression through mechanisms involving altered sensory input [11,17], chronic stress [23], neuroinflammation [51,52], and neuromodulatory dysregulation [13,14].

A primary strength of these human epidemiological and clinical studies is their high translational relevance and large-scale validation, although their inherent limitation lies in the inability to entirely exclude socioeconomic confounders or directly observe early neuropathological initiation, as highlighted in comprehensive expert consensus reviews [58].

### 3.4. Experimental Animal Evidence and Molecular Mechanisms

To dissect specific biological pathways and establish causality, experimental animal models employ targeted masticatory manipulations, such as soft-diet (powdered) feeding, molar extraction, or the experimental reduction in occlusal support. These observations are highly consistent with experimental findings demonstrating that reduced masticatory activity directly impairs hippocampal neurogenesis, synaptic plasticity, and spatial learning performance [7,10,16]. Mechanistically, animal models have shown that masticatory hypofunction alters hippocampal BDNF–TrkB signaling, with reduced TrkB expression and impaired downstream signaling reported in several models. Furthermore, reduced masticatory input suppresses key synaptic-plasticity-related proteins, including synaptophysin and postsynaptic density protein 95, and is associated with impaired neurogenesis, dendritic remodeling, and synaptic dysfunction [59,60].

Collectively, these well-controlled animal studies support the concept that physiological mastication contributes to the maintenance of neural network integrity throughout life [7,10,17]. The core strength of these animal paradigms is the ability to isolate pure sensorimotor afferent loss and directly assess molecular tissue. However, they carry limitations, as tooth-extraction models can introduce potential confounding factors like transient systemic inflammation and altered craniofacial biomechanics, while soft-diet models may inherently modify nutritional intake dynamics.

### 3.5. Human Evidence and the Oral–Brain Axis

**Figure 2:**
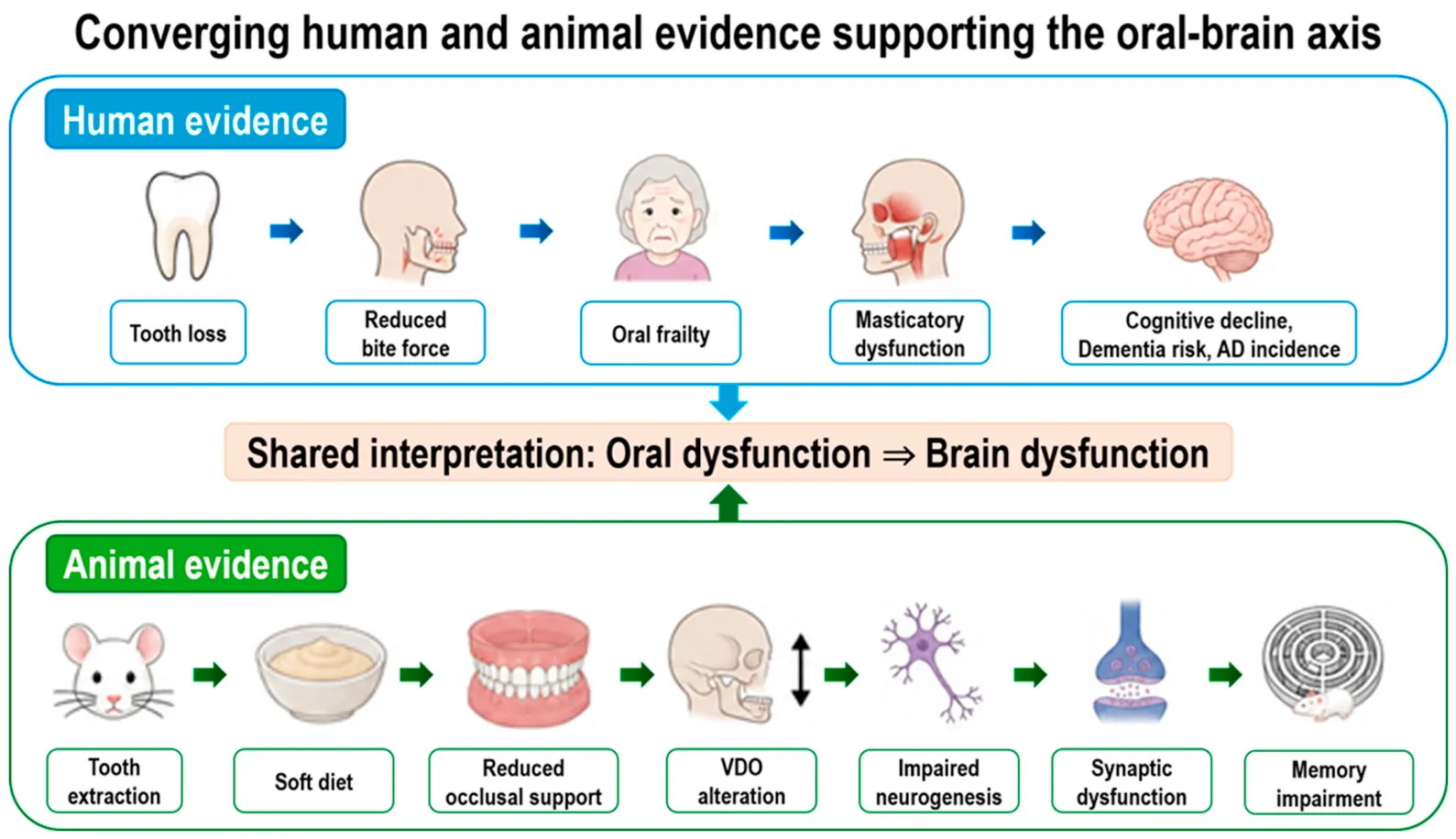
Converging human and animal evidence supporting the oral–brain axis. Clinical studies indicate that tooth loss, reduced bite force, reduced occlusal support, and masticatory dysfunction are associated with cognitive decline, dementia risk, and Alzheimer’s disease. Experimental studies demonstrate that tooth extraction, soft-diet feeding, reduced occlusal support, and alterations in the vertical dimension of occlusion (VDO) impair neurogenesis, synaptic plasticity, and memory performance. Together, these findings support the concept of an oral–brain axis linking oral dysfunction with adverse neurological outcomes.

Collectively, epidemiological, clinical, and longitudinal evidence supports a significant relationship between oral function and brain health [5,7,8,9]. Human studies alone cannot determine whether oral dysfunction acts as a causal driver of neurodegeneration, an early manifestation of disease, or both [5,8]. Nevertheless, these findings are highly consistent with experimental observations implicating trigeminal sensory pathways, MesV vulnerability, LC-dependent stress regulation, and predictive-coding mechanisms [11,13,14,18,19,21]. Within the proposed oral–brain axis framework, oral dysfunction may therefore represent an accessible window into early network instability that develops years before overt neurodegenerative symptoms emerge [1,2,3,4]. Because oral sensory and masticatory functions are measurable, modifiable, and clinically accessible, they offer promising opportunities for early detection, risk stratification, and preventive intervention [6,7,17]. The convergence of epidemiological observations, clinical findings, and experimental animal studies supporting the oral–brain axis concept is summarized in Figure 2. 

## 4. Sensory-Gating Failure in Prodromal Parkinsonian Neurodegeneration

### 4.1. Early Sensory Dysfunction in PD

PD is increasingly recognized as a disorder of distributed neural networks rather than one confined to degeneration of nigrostriatal dopaminergic neurons [1,3,25]. Sensory abnormalities, including olfactory, gustatory, and somatosensory disturbances, frequently emerge years before motor symptoms become clinically detectable [1,3,61,62]. These early sensory deficits likely reflect dysfunction within ascending brainstem and cortical pathways involved in sensory gating, interoception, and salience processing [3,25]. According to Braak’s staging hypothesis, α-synuclein pathology may originate within peripheral sensory structures and lower brainstem nuclei before progressively ascending toward midbrain dopaminergic systems [25,63,64]. Experimental support for this concept has been provided by intranasal rotenone models, in which mitochondrial complex-I inhibition is introduced through olfactory and trigeminal routes [65,66,67]. Because sensory-processing pathways are affected before substantial nigrostriatal degeneration develops, behavioral abnormalities emerge while locomotor function remains relatively preserved [3,65,66]. This temporal dissociation provides a unique opportunity to investigate sensory-gating failure, defined as a disruption in the neural mechanisms responsible for filtering, prioritizing, and integrating incoming sensory information during the prodromal phase of PD [1,3,25].

Furthermore, early oral dysfunction in PD may not only precede classical motor signs but also interact continuously with the broader non-motor symptom burden [1,3]. Accumulating clinical insights suggest that disturbances within the oral–brain axis—such as dysphagia, sialorrhea, and altered gustatory processing—frequently co-occur and synergize with other prodromal non-motor features, including sleep disturbances, autonomic dysfunction, anxiety, and depression [3,25]. Rather than being isolated secondary manifestations, persistent disturbances in oral sensorimotor networks and associated brainstem integration may exacerbate the overall clinical severity of these non-motor proteinopathies throughout the disease course [25,63].

### 4.2. Menthol Sensitivity as a Functional Biomarker

Among the earliest sensory abnormalities observed in prodromal Parkinsonian models is reduced responsiveness to menthol stimulation [65,68]. Menthol activates peripheral transient receptor potential melastatin-8 receptors [69,70] while simultaneously engaging central trigeminal sensory pathways [11]. In intranasal rotenone models, menthol sensitivity is significantly reduced during the prodromal stage despite the absence of overt motor deficits [65,66,68]. Because these behavioral abnormalities exceed the degree of detectable peripheral structural damage, the deficit is unlikely to be explained solely by receptor loss [65,68]. Instead, it may reflect dysfunction of central sensory-gating mechanisms [3,25]. Sensory gating serves to prevent excessive transmission of irrelevant sensory information and enables efficient allocation of neural processing resources [71,72]. When this function becomes impaired, information-processing efficiency declines and abnormal behavioral responses may emerge despite relatively preserved peripheral sensory structures [72,73]. Reduced menthol responsiveness may therefore represent a sensitive, inexpensive, and non-invasive functional biomarker of early trigeminal-cortical sensory-gating dysfunction [3,72,74].

### 4.3. Insular Dysfunction and Sodium-Channel Mechanisms

The insular cortex is a key hub for integrating gustatory, trigeminal, autonomic, and interoceptive information and plays a central role in the generation of subjective sensory experiences [12,75]. Experimental studies have demonstrated that rotenone exposure induces impairments in inhibitory synaptic transmission, synaptic plasticity, and conditioned taste-aversion learning within insular cortical circuits before the appearance of major motor abnormalities [65,76].

Recent electrophysiological findings have suggested a novel mechanism underlying menthol-related sensory-gating deficits. Although menthol has traditionally been viewed primarily as a TRPM8 agonist [69,70], patch-clamp recordings indicate that menthol may also suppress neuronal excitability by facilitating voltage-dependent sodium-channel inactivation, thereby reducing action-potential generation independently of canonical TRP-channel signaling [77,78]. Voltage-gated sodium channels play essential roles in action-potential initiation, neuronal synchronization, and network oscillations [79,80]. Even modest reductions in channel availability can impair sensory filtering and disrupt information processing within cortical circuits [72,81]. Consequently, rotenone-induced sodium-channel dysfunction may provide a cellular mechanism linking sensory-gating failure to early behavioral abnormalities observed during prodromal PD [76,78,81].

### 4.4. Clinical Implications

**Figure 3:**
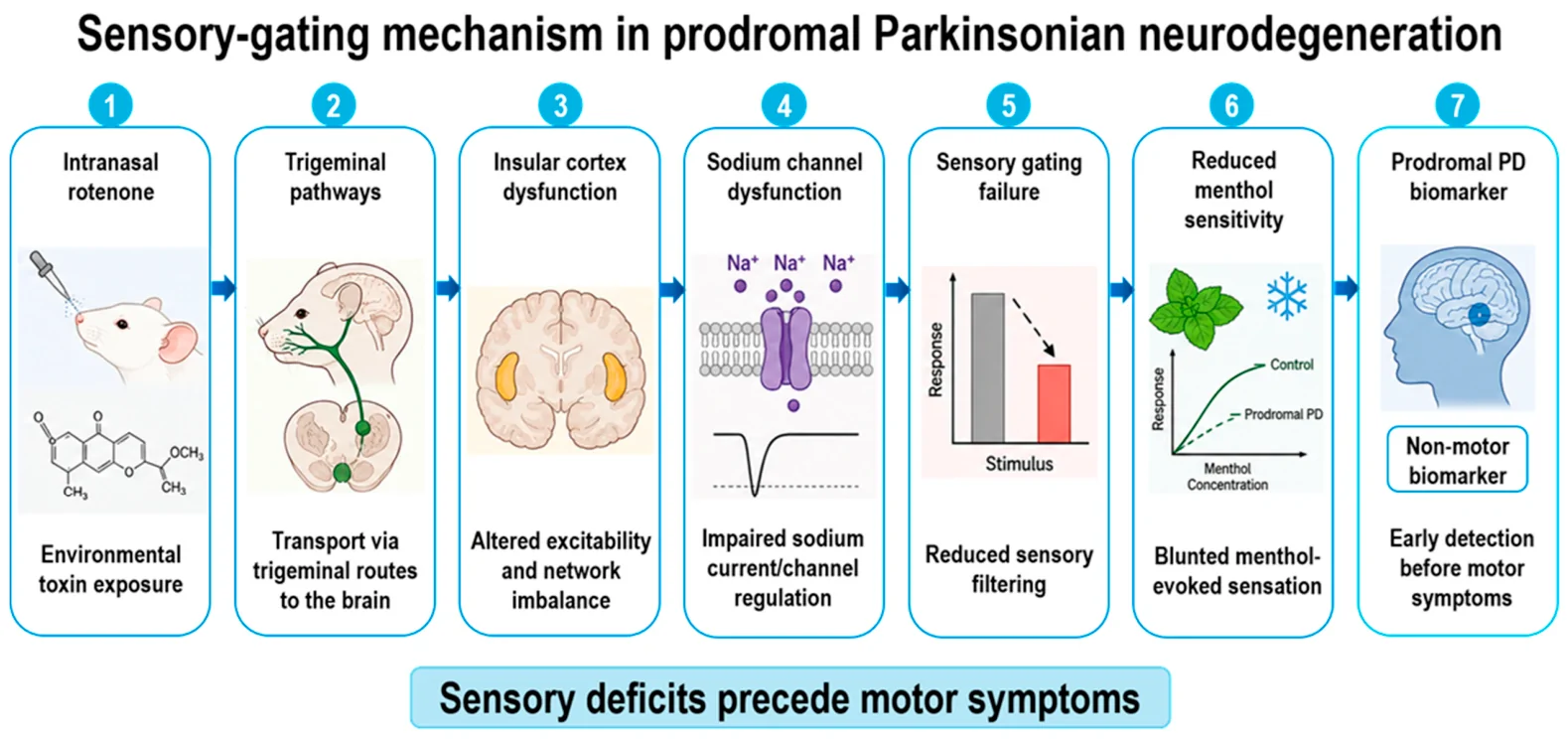
Sensory-gating dysfunction as a prodromal mechanism in Parkinsonian neurodegeneration. Intranasal rotenone exposure affects trigeminal sensory pathways and is associated with dysfunction of insular cortical circuits, leading to impaired sodium-channel-related processing and sensory-gating failure. Reduced responsiveness to menthol stimulation may serve as a functional biomarker of prodromal PD before the onset of characteristic motor symptoms.

The emergence of trigeminal and insular sensory abnormalities during the prodromal phase of PD has important translational implications [1,3,25]. Menthol-based chemosensory testing is inexpensive, non-invasive, repeatable, and potentially suitable for large-scale screening, making it an attractive candidate for the early detection of Parkinsonian neurodegeneration [65,68]. Furthermore, sensory-gating failure repeatedly engages stress-responsive neural circuits, particularly the LC, which regulates vigilance, arousal, adaptive behavior, and attentional allocation [13,14]. Persistent sensory mismatch may therefore contribute to transforming localized network instability into broader stress-system dysregulation and neuromodulatory imbalance [13,14,18,19]. Within the oral–brain axis framework, prodromal PD provides a useful example of how trigeminal sensory dysfunction, insular cortical channelopathy, and LC-dependent stress activation may converge to generate early neurodegenerative vulnerability [3,11,12,13,14]. Collectively, these findings suggest that sensory-gating failure may represent more than an early symptom and could contribute to mechanisms underlying Parkinsonian neurodegeneration [3,14,25,72,81]. A schematic overview of the proposed pathway linking trigeminal sensory dysfunction, insular channelopathy, and impaired sensory gating during the prodromal phase preceding motor symptom onset is presented in Figure 3.

Although the current discussion has focused primarily on PD, similar mechanisms may also be relevant to atypical parkinsonian syndromes, including multiple system atrophy, progressive supranuclear palsy, and corticobasal degeneration. Increasing evidence suggests that neuroinflammatory processes contribute substantially to disease progression across both typical and atypical parkinsonian disorders [31,32,82]. Because the oral–brain axis framework incorporates chronic stress signaling, LC dysfunction, neuroimmune activation, and sensory-processing abnormalities, it may provide a broader mechanistic perspective applicable beyond classical PD [3,11,12,13,14].

While direct evidence linking oral dysfunction to atypical parkinsonian syndromes remains limited, assessment of oral sensory function, mastication, and trigeminal network integrity may provide useful avenues for future investigation [1,3,82]. More broadly, the oral–brain axis hypothesis raises the possibility that oral sensorimotor dysfunction and associated neuroimmune responses represent common modifiers of neurodegenerative vulnerability across multiple proteinopathies rather than disease-specific features confined to PD. This perspective may help integrate observations across disorders characterized by varying contributions of α-synuclein, tau, and related proteinopathies.

## 5. Neuroinflammation and Glial Mechanisms

### 5.1. Neuroinflammation as a Common Pathway

Neuroinflammation is now recognized as a central pathological feature of AD, PD, and numerous other neurodegenerative disorders [31,32]. Although inflammatory responses were historically viewed as secondary consequences of neuronal degeneration, accumulating evidence indicates that neuroinflammation actively contributes to disease initiation and progression [31]. Microglia and astrocytes continuously monitor neuronal activity and maintain tissue homeostasis within the CNS [33,34]. Under chronic pathological conditions, however, these glial cells undergo functional reprogramming that promotes inflammatory signaling, synaptic dysfunction, and neuronal vulnerability [31,32].

Within the oral–brain axis framework, neuroinflammation may represent a critical intermediary linking chronic oral sensorimotor dysfunction, stress-system dysregulation, and progressive neurodegeneration. Persistent sensory mismatch, particularly involving MesV-centered circuits, may contribute to neuroimmune activation [83,84]. Neuroinflammation and chronic stress are increasingly recognized as mutually reinforcing processes [36]. Sustained activation of stress-responsive neural circuits, particularly those involving the LC, enhances norepinephrine release and alters glial reactivity [34]. Although acute noradrenergic signaling may exert anti-inflammatory effects, prolonged stress exposure promotes maladaptive neuroimmune responses characterized by microglial priming, astrocytic activation, and increased susceptibility to inflammatory stimuli [36]. Consequently, chronic stress generated by persistent oral sensorimotor instability may amplify neuroinflammatory vulnerability long before overt neurodegenerative pathology becomes clinically apparent.

### 5.2. Microglial Activation

Microglia serve as the resident immune cells of the CNS and rapidly respond to abnormal neuronal activity, protein aggregates, tissue injury, and stress-related signaling [35]. Experimental studies have demonstrated that chronic stress induces microglial activation in multiple brain regions, including the hippocampus, amygdala, and prefrontal cortex [85,86]. Beyond immediate responses, excessive or persistent activation may promote pathological synapse elimination and accelerate cognitive decline through complement-mediated synaptic pruning [87,88]. Such mechanisms are increasingly implicated in both AD and PD, representing a critical pathway linking chronic trigeminal network instability to progressive neurodegenerative vulnerability [85,89].

### 5.3. Microglial Priming as an Early Consequence of Chronic Prediction Error

Evidence from experimental animal models and histopathological studies suggests that microglial alterations occur early in the disease process, even before overt neuronal loss becomes detectable [54]. Within the framework proposed here, persistent prediction error processing driven by chronic occlusal mismatch and trigeminal sensorimotor instability may increase neuronal metabolic burden and trigger the release of stress-associated signaling molecules that prime microglia [54]. Primed microglia exhibit subtle morphological changes but remain functionally hypersensitive to subsequent stressors, protein aggregates, or aging-related pathology [54,90]. This condition lowers the threshold for exaggerated inflammatory responses and may establish a permissive neuroimmune environment that promotes neurodegenerative vulnerability long before clinical symptoms emerge [54,91]. Importantly, this primed state can be induced in the absence of overt tissue injury [92]. Altered neuronal firing patterns, network oscillations, or metabolic demand release pro-inflammatory cytokines, ATP, HMGB1, and other danger-associated molecular patterns [35,86,92]. Within the MesV-centered predictive-coding framework, this continuous processing of sensorimotor prediction errors imposes chronic energetic stress on trigeminal circuits, providing a direct mechanistic pathway linking oral sensorimotor dysfunction to chronic neuroimmune activation [35,92].

### 5.4. Astrocytes and Network Homeostasis

Astrocytes are increasingly recognized as active regulators of neuronal communication rather than passive support cells [93]. These cells play critical roles in extracellular potassium buffering, glutamate uptake, metabolic coupling, and neurovascular regulation [94,95]. Under chronic stress and inflammatory conditions, astrocytes undergo reactive transformation characterized by altered neurotransmitter regulation, impaired metabolic support, and increased production of inflammatory mediators [96]. Reactive astrocytes can disrupt excitation–inhibition balance, impair synaptic homeostasis, and facilitate excitotoxic neuronal injury [97]. Crucially, astrocytes are major regulators of extracellular glutamate homeostasis through excitatory amino acid transporters [94,97]. Impaired glutamate clearance results in prolonged activation of glutamate receptors, increased intracellular calcium loading, and enhanced vulnerability to excitotoxic injury [97]. Because precise control of membrane excitability and neuronal firing patterns is essential for MesV function, such astrocytic impairment may significantly alter sensory processing, sensorimotor integration, and sensory-gating mechanisms, further exacerbating channel dysfunction and destabilizing trigeminal networks [94,97].

### 5.5. Disease-Associated Microglia and Neurodegenerative Vulnerability

Recent single-cell transcriptomic studies have identified disease-associated microglia, a specialized microglial phenotype observed in AD and related neurodegenerative disorders [98]. DAM phenotypes are characterized by profound transcriptional reprogramming involving lipid metabolism, phagocytosis, and inflammatory signaling pathways [98,99]. Although DAM may initially contribute to the clearance of pathological protein aggregates, prolonged activation is associated with enhanced inflammatory signaling, synaptic loss, neuronal dysfunction, and disease progression [99,100]. Within the oral–brain axis framework, chronic trigeminal network instability, particularly involving MesV dysfunction and LC-dependent stress signaling, may promote early transitions toward DAM-like phenotypes. While transitions toward DAM phenotypes occur remarkably early during disease progression and often precede substantial neuronal loss, maladaptive neuroimmune responses may actively contribute to disease initiation rather than merely reflecting downstream consequences of neurodegeneration [54,98,99]. Although direct evidence remains unavailable, this possibility provides a biologically plausible mechanism through which chronic oral sensorimotor disturbances could influence long-term neurodegenerative risk.

### 5.6. Reactive Astrocytes and Synaptic Failure

A growing body of evidence indicates that activated microglia can induce the formation of neurotoxic A1 reactive astrocytes through cytokine-mediated signaling pathways [101]. These astrocytes exhibit impaired glutamate clearance, disrupted potassium buffering, and reduced neurotrophic support [101]. As a consequence, neuronal firing becomes destabilized and synaptic integration progressively deteriorates [97,101]. Circuits already burdened by chronic prediction error processing may be particularly susceptible to these effects. Together, microglial priming, DAM-like transitions, and astrocytic reactivity may create a neuroimmune environment that favors neurodegenerative progression [54,99,101] and may potentially influence downstream pathological processes that have been associated with AEP activation and tau pathology [102]. Astrocytes may also participate directly in proteinopathic processes [97]. Recent evidence suggests that inflammatory signaling and oxidative stress alter lysosomal function and protease activity within astrocytes, potentially affecting proteolytic pathways that may include AEP-related mechanisms. Although direct evidence linking oral sensorimotor dysfunction to astrocytic AEP activation is currently lacking, chronic glial activation may represent an intermediary connecting network instability with pathological tau processing, although the specific contribution of astrocytic AEP remains speculative. While the precise contribution of astrocytic AEP remains speculative at this stage, it highlights a critical avenue for future interdisciplinary research.

### 5.7. Neuroinflammation as the Missing Link Between MesV Dysfunction and Proteinopathy

One of the major unanswered questions in the oral–brain axis is how localized sensorimotor dysfunction can ultimately influence molecular pathways associated with neurodegeneration. Neuroinflammation may provide this missing link [54]. Specifically, as described in Section 5.3, these neuroinflammatory alterations involve microglial priming and astrocytic reactivity [54,101]. These changes have been implicated in proteolytic processes associated with proteinopathy, potentially involving AEP among other candidate mechanisms [103]. Experimental animal models and biochemical studies indicate that AEP activation is enhanced under conditions associated with aging, tissue acidosis, oxidative stress, and chronic neuroinflammatory states [102,103], suggesting that glial activation may indirectly influence molecular pathways implicated in tau pathology [101,102]. Because the specific LC-linked molecular cascade involving AEP activation and tau cleavage is discussed in detail in Section 6, its detailed explanation is omitted here. Taken together, microglial priming, DAM-like transitions, reactive astrocytosis, and chronic neuroinflammation may represent an important intermediate process linking oral sensorimotor dysfunction and LC-related stress signaling to downstream proteinopathic changes [54,99,101,102]. In this framework, neuroinflammation may act not only as a consequence of neural dysfunction but also as a potential amplifier through which persistent network instability could contribute to progressive neurodegenerative pathology [54,99,102].

### 5.8. Limitations and Alternative Interpretations

Although the oral–brain axis framework integrates diverse observations from dentistry, neuroscience, stress biology, and neurodegenerative disease research, direct causal evidence linking chronic oral sensorimotor mismatch to AEP-mediated tau pathology remains limited. Several components of the proposed pathway, particularly the transitions from MesV dysfunction to LC dysregulation and from neuroimmune activation to AEP-dependent proteinopathy, remain hypothetical and require experimental validation. Future studies combining MesV electrophysiology, in vivo imaging of LC activity, neuroimmune profiling, and longitudinal cognitive assessment will be necessary to determine whether these mechanisms contribute causally to neurodegenerative disease progression. Nevertheless, the framework generates testable predictions and provides a useful model for investigating how chronic disturbances in oral sensorimotor homeostasis may interact with stress-responsive and neuroimmune pathways to influence brain aging and neurodegenerative vulnerability.

To validate this framework, a critical analysis of current research models is essential. Traditional methods like soft-diet feeding and molar extraction successfully link reduced mastication to cognitive decline, but they introduce major confounding factors such as systemic inflammation, pain, and nutritional changes [7,10,16,17,38]. To isolate pure sensory effects, recent models utilizing VDO alterations preserve dentition and nutrition, yet direct in vivo electrophysiological evidence of long-term MesV-LC destabilization remains limited [17,39,50]. Similarly, the intranasal rotenone model effectively captures prodromal Parkinsonian sensory-gating deficits, but its findings are constrained by systemic, toxin-specific effects [65,66,67,68]. Finally, while longitudinal human cohorts provide invaluable clinical relevance associating oral frailty with dementia risk, they are fundamentally limited by residual systemic confounders and cannot definitively establish causality [5,8,9]. Acknowledging these model-specific limitations is crucial for designing future interdisciplinary studies.

## 6. Chronic Stress and LC-AEP Amplification

### 6.1. Developmental and Oral Origins of Stress Vulnerability

Chronic stress functions as a central biological amplifier that may transform localized trigeminal dysfunction into widespread neurodegenerative vulnerability [37]. Although trigeminal sensorimotor disturbances and sensory-gating abnormalities may emerge early, their pathological impact likely depends on the responsiveness of stress-regulatory systems [37,104]. A critical component of this vulnerability may originate during early postnatal development [91]. During the transition from suckling to mastication, trigeminal circuits undergo extensive activity-dependent maturation [105]. Exposure to stress during this critical developmental period, particularly elevated glucocorticoid signaling, may alter the development and long-term functional properties of trigeminal sensorimotor networks [91,105]. Experimental studies further suggest that early-life stress can produce lasting impairments in masticatory behavior and sensory responsiveness that persist into adulthood [105]. In later life, persistent oral dysfunctions, including occlusal instability, masticatory insufficiency, and chronic sensory mismatch, may serve as continuous sources of low-grade sensorimotor stress [106]. Unlike acute injury, these disturbances impose sustained demands for adaptive recalibration, repeatedly engaging stress-responsive neural circuits in the absence of overt tissue damage [107]. Over time, this chronic engagement may promote maladaptive stress phenotypes and increase vulnerability to neurodegenerative disease [37,91].

### 6.2. Sleep Bruxism and OSA as Stress Amplifiers

Two highly prevalent clinical conditions, sleep bruxism and OSA, may further amplify stress-dependent signaling within the oral–brain axis [23,108]. Sleep bruxism, characterized by rhythmic masticatory muscle activity (RMMA) during light non-rapid eye movement sleep [108,109], is increasingly viewed as a manifestation of altered brain-state regulation and sympathetic activation rather than a purely dental disorder [23,108]. Chronic stress and anxiety increase RMMA frequency [23], producing repeated proprioceptive and nociceptive activation of trigeminal pathways that may overload MesV-centered sensorimotor circuits. OSA contributes an additional physiological burden through intermittent hypoxia, oxidative stress, sleep fragmentation, and autonomic dysregulation [110]. Experimental studies have demonstrated that intermittent hypoxia enhances sensory excitability and TRPV1-dependent signaling [110], thereby amplifying peripheral sensory signaling and stress-related neural activation. Together, sleep bruxism and OSA may function as complementary mechanical and physiological stress amplifiers converging on central stress networks and promoting neuroinflammatory signaling.

### 6.3. The LC as the Central Hub

Beyond its established role in arousal and adaptive behavior, the LC occupies a strategic position within the proposed oral–brain axis because of its extensive projections to limbic and cortical networks [24,37]. Through these widespread connections, the LC functions as a central integrative hub that translates sensory mismatch and emotional stress into global neuromodulatory responses. Importantly, the LC is among the earliest sites affected by pathological changes in both AD and PD [111,112,113], suggesting that persistent oral sensorimotor disturbances may influence LC activity during prodromal disease stages. Stress-related disruption of LC homeostasis has also been associated with altered noradrenergic signaling and increased vulnerability to downstream neurodegenerative processes [29,30]. Together, these observations highlight the LC as a biologically plausible upstream regulator within the oral–brain axis.

### 6.4. The LC-AEP Pathway

The discovery of the AEP pathway provides a mechanistic link between chronic stress-induced LC dysregulation and neurodegenerative proteinopathy [102]. Under physiological conditions, LC neurons maintain stable firing through multiple autoregulatory mechanisms, but chronic stress promotes sustained LC hyperactivity, neuronal remodeling, and altered noradrenergic signaling [37,104]. Experimental animal and molecular studies have identified the LC-AEP pathway as a potential bridge between stress and tau pathology. Kang et al. demonstrated that DOPEGAL, a norepinephrine metabolite selectively generated in LC noradrenergic neurons, activates AEP, inducing pathological tau cleavage, LC neurodegeneration, and propagation of tau pathology to connected forebrain regions [27,114]. Consistent with this concept, chronic stress impairs LC autoinhibitory mechanisms and increases AEP activity [29], whereas sleep-fragmentation-associated noradrenergic activation promotes AEP-dependent LC degeneration and downstream proteinopathic changes [30]. Collectively, these findings suggest that persistent LC dysregulation may facilitate AEP activation and pathological tau processing, providing a molecular framework through which chronic oral sensorimotor mismatch could contribute to neurodegenerative progression.

### 6.5. Noradrenergic Destabilization of Trigeminal Networks

Excessive LC activity propagates downstream through noradrenergic volume transmission [37]. Unlike classical synaptic transmission, norepinephrine diffuses through the extracellular space, modulating neuronal excitability across broad regions of the brainstem and forebrain [24]. Under chronic stress, dysregulated noradrenergic signaling alters membrane excitability, impairs proprioceptive encoding, amplifies sensory prediction errors, and reinforces maladaptive motor output [37]. Over time, these changes may progressively destabilize MesV-centered sensorimotor networks, potentially creating a self-reinforcing feedforward loop in which trigeminal dysfunction contributes to altered LC activity, whereas altered LC output may further exacerbate trigeminal network instability. This reciprocal interaction may represent a biologically plausible mechanism linking chronic oral sensorimotor dysfunction to broader neuromodulatory dysregulation and neurodegenerative vulnerability. In addition, sustained LC dysregulation may increase engagement of the LC-AEP pathway, providing a potential molecular route through which chronic stress-related neuromodulatory imbalance contributes to tau pathology and progressive neurodegeneration [29,30].

### 6.6. Unified Stress-Amplification Model

**Figure 4:**
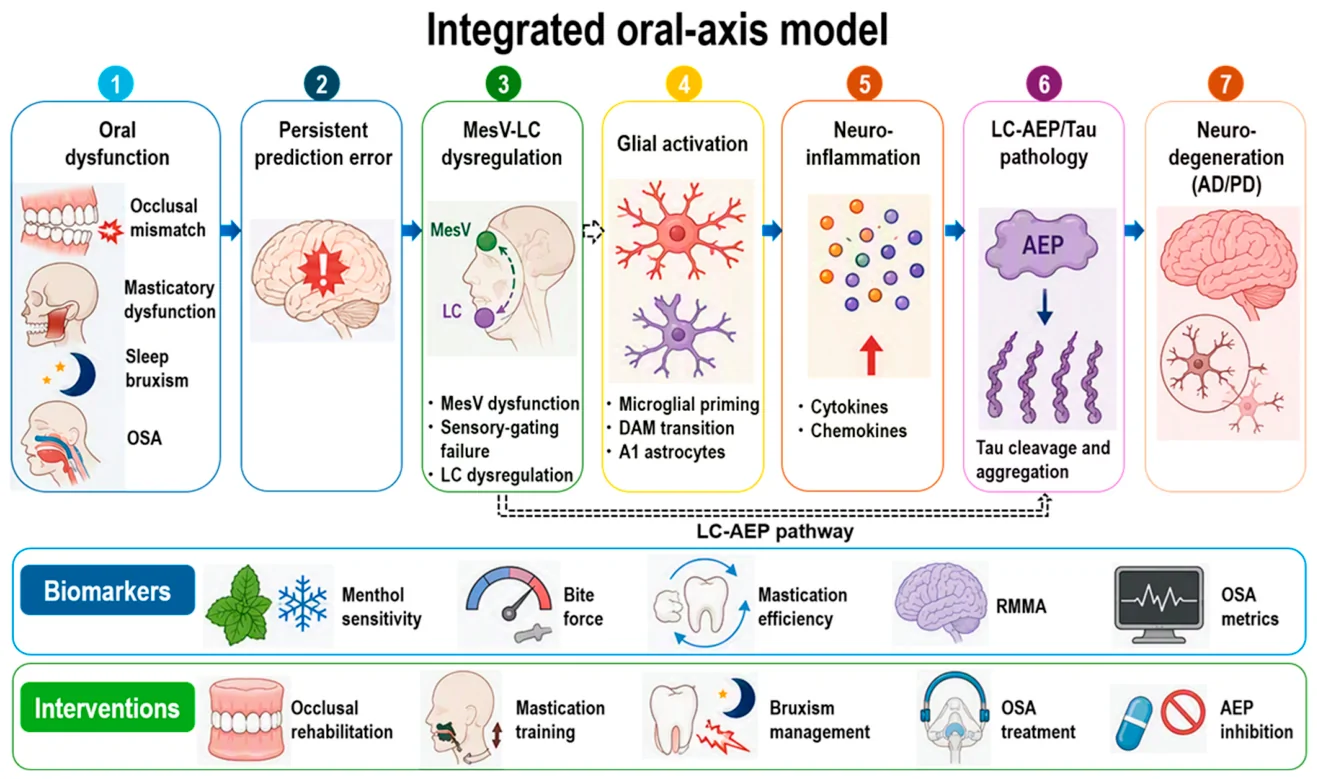
Integrated oral–brain axis model linking oral dysfunction to neurodegeneration. Integrated conceptual model of the oral–brain axis. Oral conditions including occlusal mismatch, masticatory dysfunction, sleep bruxism, and obstructive sleep apnea (OSA) may generate persistent sensorimotor prediction errors and disrupt MesV-centered networks. Chronic network instability may engage LC-dependent stress systems and promote neuroimmune activation, including microglial priming, reactive astrocytosis, and DAM-like transitions. A proposed LC-AEP pathway links LC dysregulation to AEP activation, tau cleavage, and aggregation, whereas neuroinflammatory processes may further amplify neurodegenerative cascades. Through these interacting pathways, chronic oral sensorimotor dysfunction may contribute to increased neurodegenerative vulnerability. The proposed LC-AEP pathway is based on experimental animal studies linking LC noradrenergic dysfunction to AEP activation and tau pathology. Proposed biomarkers include menthol sensitivity, bite force, masticatory efficiency, rhythmic masticatory muscle activity (RMMA), and OSA-related measures. Potential interventions include oral rehabilitation, management of sleep-related oral disorders, and modulation of neuroimmune and AEP-related pathways. Solid arrows indicate relationships supported by experimental and clinical evidence, whereas dashed arrows indicate proposed pathways that remain to be fully validated.

Based on the evidence reviewed above, this review proposes that chronic oral sensorimotor mismatch may generate persistent prediction error signaling within trigeminal networks. Repeated activation of MesV-centered circuits may interact with LC-related stress systems, contributing to neuromodulatory imbalance, neuroimmune activation, and neuroinflammation. Through activation of the proposed LC-AEP pathway, sustained LC dysregulation may promote AEP activation, pathological tau cleavage, and propagation of tau pathology [27,29,30,114]. These processes may ultimately converge on progressive neurodegenerative cascades characteristic of AD and PD. This provides a conceptual framework for how localized oral sensorimotor disturbances might propagate into brain-wide pathological processes. The complete oral–brain axis model is summarized in Figure 4.

## 7. Clinical Translation and Future Directions

### 7.1. Biomarkers and Therapeutic Opportunities

The oral–brain axis framework shifts the paradigm of neurodegenerative disease management by identifying therapeutic and diagnostic targets that operate upstream of irreversible neuronal loss. Among potential therapeutic strategies, inhibition of AEP represents a promising disease-modifying approach because it may interrupt stress-dependent tau cleavage and subsequent protein aggregation [102,103]. In parallel, modulation of ion channels involved in trigeminal excitability, including TASK1 leak potassium channels and voltage-gated sodium channels, may help restore physiological activity within destabilized MesV-centered networks and thereby improve sensorimotor homeostasis [44,115,116].

Beyond pharmacological interventions, oral rehabilitation may function as a form of central neuromodulation. Restoration of oral sensorimotor homeostasis through occlusal adjustment, masticatory training, management of temporomandibular disorders, and the use of oral appliances for sleep bruxism and OSA may reduce abnormal sensory afferent input to the brainstem [7,16]. By decreasing chronic activation of trigeminal pathways, these interventions could potentially attenuate LC hyperactivity and limit the activation of downstream neurodegenerative cascades during prodromal stages of disease. The oral–brain axis also provides a novel framework for biomarker development. Quantitative assessment of oral sensory and motor function, including trigeminal chemosensory testing such as menthol sensitivity [68], measures of masticatory efficiency, bite-force assessment, and monitoring of RMMA [108], may offer a low-cost, non-invasive, and repeatable platform for detecting neural network instability before the onset of overt cognitive or motor symptoms.

### 7.2. Establishing Causality and Interdisciplinary Research

A major challenge for future research is to determine whether oral dysfunction acts as a causal contributor to neurodegenerative disease, an early manifestation of underlying pathology, or both. Addressing this issue will require longitudinal, multidisciplinary investigations integrating oral physiology, neuroscience, neuroimaging, and molecular biomarker research.

One important priority is the development of prospective interventional studies. Most available evidence is observational and cannot fully exclude reverse causation. Studies examining whether correction of occlusal instability, restoration of masticatory function, treatment of sleep bruxism, or management of OSA modifies trajectories of cognitive decline would provide critical information regarding causality. A second priority is the identification of biomarkers of oral–brain axis dysfunction. Quantitative measures of bite force, masticatory efficiency, occlusal stability, and trigeminal sensory performance may provide inexpensive and repeatable assessments of network integrity. In particular, menthol sensitivity testing may represent a practical measure of trigeminal–insular sensory-gating function during prodromal disease stages. Future studies should determine whether oral biomarkers improve prediction of cognitive decline when combined with established neurodegenerative biomarkers [1,2,3,4].

Advanced neuroimaging and mechanistic studies will also be essential. Imaging of LC integrity, neuroinflammation, and hippocampal networks may clarify temporal relationships among trigeminal dysfunction, stress system activation, and neurodegenerative pathology. In parallel, experimental studies are needed to validate the proposed MesV-LC-neuroinflammation-AEP pathway and determine whether chronic oral sensorimotor dysfunction directly contributes to disease progression [29,39,50,98,99,100,101,102]. Collectively, these approaches may help transform the oral–brain axis from a theoretical framework into a clinically actionable model for early detection, risk stratification, prevention, and therapeutic intervention in neurodegenerative disorders.

Another critical avenue for future investigation is determining whether the oral–brain axis differentially impacts specific disease subtypes and phenotypic variants. In AD, classical amnestic and non-amnestic cortical phenotypes may exhibit distinct patterns of LC and network vulnerability [26]. Similarly, the progression of typical PD differs fundamentally from that of atypical parkinsonian syndromes such as multiple system atrophy or progressive supranuclear palsy [1,3,25]. While direct evidence linking oral sensorimotor dysfunction to subtype-specific neurodegenerative evolution remains insufficient, quantitative oral metrics—when integrated with neurological examinations, neuroimaging, and molecular biomarkers—may eventually contribute to phenotypic stratification and personalized risk profiling before advanced multi-system degeneration occurs [1,3].

Achieving these goals will require bridging the long-standing divide between dentistry and neurology. Because the trigeminal system occupies a unique position at the interface between oral function and brain regulation, future advances are likely to emerge from collaborative efforts that integrate dentistry, neuroscience, sleep medicine, and neurology. Such an interdisciplinary approach may ultimately establish the oral–brain axis as a clinically actionable framework for the early detection, prevention, and treatment of neurodegenerative diseases.

## 8. Conclusions

Accumulating epidemiological, clinical, and experimental evidence indicates that oral function is closely linked to brain health and neurodegenerative vulnerability. Tooth loss, impaired mastication, reduced occlusal support, and trigeminal sensory dysfunction are consistently associated with cognitive decline, while experimental studies demonstrate that disruption of oral sensorimotor activity can adversely affect hippocampal function, synaptic plasticity, and learning. Together, these findings suggest that oral dysfunction may represent more than a secondary consequence of aging or neurological disease.

This review proposes a unified oral–brain axis framework in which chronic disturbances of oral sensorimotor homeostasis generate persistent prediction error signaling within MesV-centered trigeminal networks, recruit LC-dependent stress pathways, and promote neuroimmune activation. Although several components of this model remain to be experimentally validated, it provides a testable framework linking dentistry and neuroscience. Future studies should determine whether oral dysfunction acts as a causal driver of neurodegeneration, an early biomarker, or both. If validated, the oral–brain axis may offer new opportunities for early detection, risk stratification, prevention, and intervention in neurodegenerative disorders.

## Data Availability

No new data were created or analyzed in this study.

## Abbreviations

AD	Alzheimer’s disease
AEP	Asparagine endopeptidase
CNS	Central nervous system
DOPEGAL	3,4-Dihydroxyphenylglycolaldehyde
LC	Locus coeruleus
MesV	Mesencephalic trigeminal nucleus
OSA	Obstructive sleep apnea
PD	Parkinson’s disease
RMMA	Rhythmic masticatory muscle activity
TASK	TWIK-related acid-sensitive K^+^ channel
VDO	Vertical dimension of occlusion
